# The Roles and Comparison of Rigid and Soft Tails in Gecko-Inspired Climbing Robots: A Mini-Review

**DOI:** 10.3389/fbioe.2022.900389

**Published:** 2022-07-15

**Authors:** Guangyuan Zang, Zhendong Dai, Poramate Manoonpong

**Affiliations:** ^1^ Institute of Bio-inspired Structure and Surface Engineering, College of Mechanical and Electrical Engineering, Nanjing University of Aeronautics and Astronautics, Nanjing, China; ^2^ Bio-inspired Robotics and Neural Engineering Lab, School of Information Science and Technology, Vidyasirimedhi Institute of Science and Technology, Rayong, Thailand

**Keywords:** gecko locomotion, bio-inspired tails, climbing robots, soft material, biomimetic design

## Abstract

Geckos use millions of dry bristles on their toes to adhere to and rapidly run up walls and across ceilings. This has inspired the successful development of dry adhesive materials and their application to climbing robots. The tails of geckos also help realize adaptive and robust climbing behavior. Existing climbing robots with gecko-inspired tails have demonstrated improved locomotion performance. However, few studies have focused on the role of a robot’s gecko-inspired tail when climbing a sloped surface and its effects on the overall locomotion performance. Thus, this paper reviews and analyzes the roles of the tails of geckos and robots in terms of their climbing performances and compares the advantages and disadvantages of robots’ tails made of rigid and soft materials. This review could assist roboticists decide whether a tail is required for their robots and which materials and motion types to use for the tail in order to fulfill their desired functions and even allow the robots to adapt to different environments and tasks.

## 1 Introduction

Bio-inspired robotics uses nature as the inspiration for the design of robotic systems that perform similar to biological systems (Allen (1999); Yang et al. (2018); Pfeifer et al. (2007)). Animal locomotion (e.g., jumping, swimming, flying, crawling, walking, and climbing) has been studied and translated into robotic design principles to create advanced robots capable of navigating in different environments, similar to animals. For example, jumping robots (Armour et al. (2007); Mo et al. (2019, 2020)), inspired by kangaroos, locusts, and frogs, and walking robots (Koh et al. (2010); Manoonpong et al. (2021); Billeschou et al. (2020); Lee et al. (2020)), inspired by centipedes, insects, and dogs, have been studied and developed for traversing uneven or rough terrain. Swimming robots (Ryuh (2009); Ko et al. (2012); Ijspeert et al. (2007)), inspired by fish, jellyfish, and salamanders, have been designed for underwater tasks. Flying robots (Phan et al. (2020); Wenfu et al. (2022); Ramezani et al. (2016)), inspired by insects, birds, and bats, have been built for rescue operations, monitoring, and goods delivery (Huang and Savkin (2018)). Crawling robots (Khan et al. (2022); Yamamoto et al. (2018); Rozen-Levy et al. (2021)), inspired by inchworms and caterpillars, have been developed for branch or pipe crawling. An increasing number of bio-inspired climbing robots have also been proposed over the last few decades. These robots have wide application prospects in narrow space exploration, as well as the inspection and maintenance of pressure vessels, oil tanks, and the glass slabs of high-rise buildings (Dethe and Jaju (2014)).

For bio-inspired climbing robot development, the functional morphology and locomotion behavior of geckos, in particular dry adhesion mechanisms (Autumn and Peattie (2002); Autumn et al. (2006a); Tian et al. (2006); Autumn and Gravish (2008); Puthoff et al. (2010); Stark et al. (2012)) and gaits (Autumn et al. (2005, 2006b); Dai et al. (2011); Schultz JT. et al. (2021)), have been extensively studied. Hence, gecko-inspired climbing robots (Menon et al. (2004); Unver et al. (2006); Kim et al. (2008); Jusufi et al. (2008, 2010, 2011); Estrada et al. (2014); Haomachai et al. (2021); Shao et al. (2022)) and dry adhesive materials (Murphy et al. (2009); Bartlett et al. (2012); Glick et al. (2018); Suthisomboon et al. (2021)) have rapidly been developed. They can adapt to different substrate surfaces (Kim et al. (2008)) and even carry 100x their own weight (e.g., 9g climber (Hawkes et al. (2015))). However, tailless gecko-inspired robots may still be unable to climb on slippery, complex, or steep terrain. Moreover, no synthetic adhesive material can fully capture the desirable properties of a gecko’s foot to enable stable and efficient climbing (Cutkosky (2015)). Adding gecko-inspired tails to robots can significantly improve their climbing because tails play an important role in the locomotion of most mammals and vertebrates (Nabeshima et al. (2019)). These improvements will be discussed in detail later (see Section 2).

**FIGURE 1:**
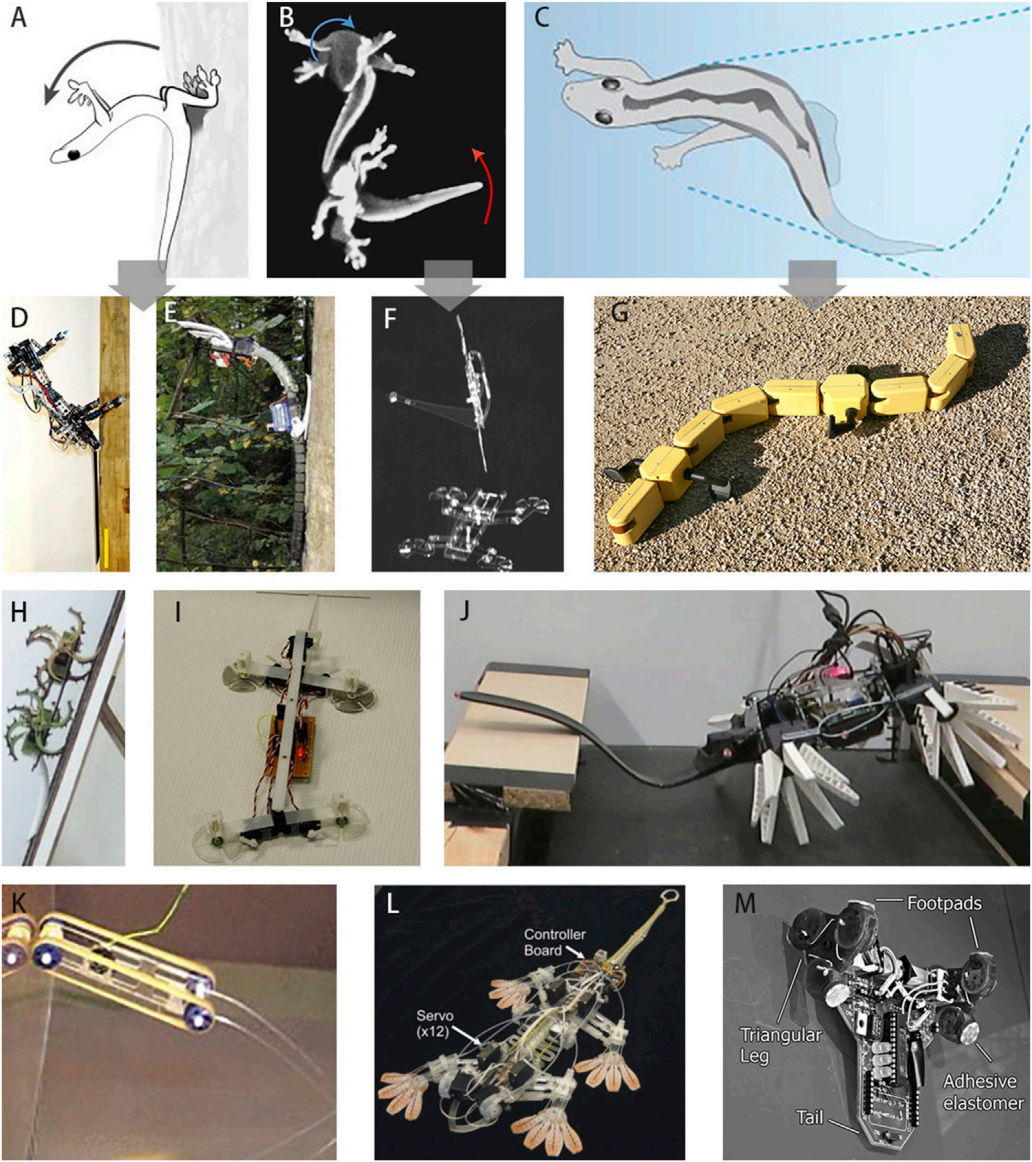
A review of the various studies and models used to assess the role of tail in locomotion. **(A)** Gecko pressing the tail tip against the wall to avoid slipping during climbing over a slippery gap (Siddall et al. (2021a)). **(B)** Gecko rotating the tail to achieve air-righting (Jusufi et al. (2008)). **(C)** The undulation of the tail assists geckos race across the water (Nirody et al. (2018)). **(D)** RiSE with an active rigid tail as an emergency fifth limb to avoid pitch-back (inspired by panel 1A) (Jusufi et al. (2008)). **(E)** RiSE with an active soft tail to assist the robot land on a tree (inspired by panel 1A) (Siddall et al. (2021a)). **(F)** Stickybot with a rotating tail to achieve air-righting (inspired by panel 1B) (Jusufi et al. (2010)). **(G)** Salamandra robotica II, a salamander inspired amphibious robot with a modular rigid tail that could undulate to help it swim (inspired by panel 1C) (Figure by A. Ijspeert, courtesy Biorobotics Laboratory, EPFL). **(H)** The slope climbing robot with a passive soft tail to assist in climbing (Siddall et al. (2021b)). **(I)** Geckobot with an active rigid tail for avoiding pitch-back (Unver et al. (2006)). **(J)** Compliant fin ray wheg robot with an active soft tail for obstacle crossing (Siddall et al. (2021b)). **(K)** Tankbot with a passive soft tail for completing plane transition (Unver and Sitti (2009)). **(L)** Stickybot I with an active tail for avoiding pitch-back (Kim et al. (2007)). **(M)** Waalbot with a fixed rigid tail to avoid pitch-back (Murphy and Sitti (2007)). Note that the first-row animal figures **(A,B,C)** correspond to the second-row robot figures **(D,E,F,G)**. All figures are reproduced with permission from respective journals.

Tails usually function as a balancing mechanism, allowing the animal to maintain balance under unstable conditions or to move rapidly and efficiently over rough terrain (Walker et al. (1998); Massaro et al. (2016)). The length of an adult salamander’s tail can reach two-thirds of its body length, indicating the importance of the tail for terrestrial locomotion and balance (Arntzen (1994)). A salamander increases the lateral undulation amplitude of its tail to increase the tail’s contact area and gain thrust while walking on a slippery substrate (Karakasiliotis and Ijspeert (2009)). Similarly, an adult gecko’s tail is almost half the length of its body (Khan (2009); Siddall et al. (2021a); Brown et al. (2010); Jusufi et al. (2010)). Pressing this long tail against a substrate helps maintain balance on slippery and complex terrain, especially on a vertical surface (Figure 1A). Moreover, the morphological features of the tail can help the gecko climb on smooth vertical surfaces, and keeled subcaudal scales can support up to approximately five times body weight (Jusufi et al. (2012)). Geckos can increase their locomotion velocity and maintain their stability on horizontal and vertical surfaces by increasing the lateral undulation amplitude and frequency of their bodies and tails (Schultz J. T. et al. (2021); Wang et al. (2020)). Geckos’ tails also allow them to effectively perform mid-air righting and turning during gliding (Autumn et al. (2005); Jusufi et al. (2008, 2010, 2011); Libby et al. (2012); Figure 1B). For example, Siddall et al. (2021c) recently investigated the role of tails in gliding locomotion using gecko experimental data with mathematical and robotic models and showed that rapid, circular tail rotations can control pitch and yaw motions in a vertical wind tunnel. Moreover, the undulation of the tail also assists a gecko in moving across water (Nirody et al. (2018); Figure 1C).

Lizards use their tails in several different ways (Libby et al. (2012); Gillis et al. (2009)). For example, the red-headed African *Agama* lizard can use its tail as an active stabilizing mechanism (Libby et al. (2012)). It raises its tail to adjust the tilt of its body and land successfully on a vertical surface when faced with a lack of footing on a horizontal slippery surface. Based on biological investigations, gecko/lizard-inspired tails were developed and applied to climbing robots to improve their locomotion performance (Menon et al. (2004); Unver et al. (2006); Kim et al. (2008); Lee et al. (2012); Liu et al. (2012)). A tail could provide a preload for a robot to reduce the required adhesive force of its front feet and prevent pitch-back (Unver et al. (2006); Menon et al. (2004); Estrada et al. (2014); Hawkes et al. (2011); Unver and Sitti (2010)). A robot with a larger tail length to body length ratio performs relatively well in terms of, e.g., obstacle traversal (Siddall et al. (2021b)), fall arrest (Menon et al. (2004); Estrada et al. (2014); Hawkes et al. (2011)), and mid-air righting (Jusufi et al. (2008, 2010); Johnson et al. (2012); Libby et al. (2016)). Some climbing robots can use their rigid tails as a support to achieve plane transition (Murphy and Sitti (2007); Murphy et al. (2011); Unver and Sitti (2010)). The aforementioned shows that gecko tails play significant roles in many locomotor scenarios, and many roboticists have applied individual functions of the tail to robots. Therefore, this review compares the advantages and disadvantages of different tail designs, with a focus on different stiffnesses (rigid and soft) and motion types (active, passive, and fixed).

## 2 Climbing Robots With Tails

Most studies have focused on developing artificial adhesive feet, which include mechanical (gripping), pneumatic (suction cups), magnetic (permanent magnet), and dry (elastomer adhesive) adhesion (Arzt et al. (2003); Kim et al. (2008); Jiang and Xu (2018); Gu et al. (2018); Schiller et al. (2019); Borijindakul et al. (2021)). Therefore, most early climbing robots were tailless (Kalouche et al. (2014); Haomachai et al. (2021); Srisuchinnawong et al. (2021)). However, animals do not rely solely on stickiness, because even the best adhesives might fail on surfaces that are critically damaged or have a layer of dust, oil, or water (Autumn et al. (2005)). Similarly, a climbing robot will inevitably experience an unexpected slip that results in an undesirable fall. Extensive research on gecko tails has revealed that geckos hold their tails down to maintain balance when their feet slip, preventing them from pitch-back (Autumn et al. (2005, 2006b); Jusufi et al. (2008)). Thus, a gecko-inspired tail has been added to some climbing robots to improve their locomotion performances (Figure 1), e.g., Geckobot (Unver et al. (2006)), Waalbot (Murphy and Sitti (2007)), Waalbot II (Murphy et al. (2011)), Tankbot-I (Unver and Sitti (2009)), Tankbot-IV (Unver and Sitti (2010)), Stickybot I (Hawkes et al. (2011)), Slope climbing robot (Siddall et al. (2021b)), and Mini-Whegs™ 7 (Daltorio et al. (2006)).

Tails can be classified into three classes according to the stiffness of the material: high, medium, and low. A high-stiffness tail cannot be deformed, whereas a medium-stiffness tail can deform under an external force but cannot completely comply or make full contact with the terrain. A low-stiffness tail is soft and complies with the terrain. To compare the roles of soft and rigid tails on robots for locomotion enhancement, we consider medium- and low-stiffness tails to be soft and high-stiffness tails to be rigid. Furthermore, the motion types of tails can be divided into three categories: active, passive, and fixed. An active tail indicates that the robot can control the tail movement in the vertical and/or lateral direction via actuator(s). A passive tail indicates that it can be moved or deformed by an external force. A fixed rigid tail cannot move or deform. As listed in Table 1, all of these tails improve the stability and maximum climbing angle of the robot (Unver et al. (2006); Kim et al. (2008); Hawkes et al. (2011); Lee et al. (2012)). The maximum climbing angle is determined with respect to the horizontal plane. The maximum slope for a slope-climbing robot with a tail length to body length ratio of 0.5 increased to 75° from 45° (tailless prototype) (Siddall et al. (2021b); Figure 1H). The maximum for Mini-Whegs™ 7, which had a tail length to body length ratio of 0.43, increased to 60° from 50° (tailless prototype), and further to 90° when the tail length to body length ratio was increased to 0.74 (Daltorio et al. (2006)). Some of these developed robots could complete plane transition after a tail was added. Tankbot-IV, which had a rigid tail could complete plane transition from the floor to a wall and from a wall to the ceiling (Unver and Sitti (2010)). However, although Tankbot-I had the same maximum climbing angle (180°), it could only complete plane transition from the floor to a wall (Unver and Sitti (2009); Figure 1K). In addition to floor-to-wall and wall-to-ceiling plane transitions, Waalbot II, which had a rigid tail, could also complete plane transition from one wall to another wall (the walls were adjacent to each other at the corner) (Murphy et al. (2011); Figure 1M).

**TABLE 1:** Characteristics of gecko-inspired robots with a tail. Preload means that the load generated by the tail to prevent pitch-back. Lizard-like gait means that two diagonal pairs of legs switch between stance and swing phases. Wheel gait means that all four “feet/wheels” are attached to the surface all the time (i.e., stance phase).

Robots		Robot Weight(g)	Tail Stiffness	Tail Length/Body Length	Tail Motion	Avoid Pitch-back		Plane Transition	Adhsive Type at Feet/wheels	Climbing Surface Type	Body Motion	Gait Pattern
						Preload	Maximum Climbing Angle					
Geckobot		100	High	0.34	Active (up/down motion)	✓	85^0^	N/A	Dry adhesive	Smooth	Fixed	Lizard-like gait
Waalbot		90	High	0.5	Fixed	✓	90^0^	Floor-to-wall	Dry adhesive	Smooth	Fixed	Wheel gait
Waalbot II		85	High	0.5	Fixed	✓	180^0^	Floor-to-wall, wall-to-wall, wall-to-ceiling	Dry adhesive	Smooth	Fixed	Wheel gait
Tankbot-IV		150	High	0.67	Active (up/down motion)	✓	180^0^	Floor-to-wall, wall-to-ceiling	Dry adhesive	Smooth, rough	Fixed	Wheel gait
Tankbot-I		90	Medium	0.57	Passive	✓	180^0^	Floor-to-wall	Dry adhesive	Smooth, rough	Fixed	Wheel gait
Stickybot-I		370	Medium	0.53	Active (up/down motion)	✓	90^0^	N/A	Dry adhesive	Smooth, rough	Fixed	Lizard-like gait
Slope climbing robot	Tailless	605	N/A	0	N/A	×	45^0^	N/A	Thermoplastic polyurethane (TPU) elastomer with spines	Smooth, rough	Fixed	Wheel gait
	Soft Tail		Low	0.5	Passive	✓	75^0^	Floor-to-slope	Thermoplastic polyurethane (TPU) elastomer with spines	Smooth, rough	Fixed	Wheel gait
Mini-Whegs ^TM^7	Tailless	87	N/A	0	N/A	×	50^0^	N/A	Dry adhesive	Smooth	Fixed	Wheel gait
	6.6 cm long tail		High	0.43	Passive	✓	60^0^	N/A	Dry adhesive	Smooth	Fixed	Wheel gait
	25 cm long tail		High	0.74	Passive	✓	90^0^	N/A	Dry adhesive	Smooth	Fixed	Wheel gait

### 2.1 Rigid Tail

The offset between a wall and load vector leads to a pitch-back moment (Hawkes et al. (2011)), which causes the front feet of a robot to detach from the wall, resulting in a pitch-back trend. This mainly causes the falling of the climbing robot. However, if a tail is added to a robot, it can generate a preload to transfer some of the load from the rear feet to the front feet, preventing the pitch-back and increasing the maximum slope for the robot (Estrada et al. (2014)). Currently, the most commonly used materials for robot tails are rigid, such as in Geckobot (Unver et al. (2006); Figure 1I), RiSE (Jusufi et al. (2008); Figure 1D), and Waalbot (Murphy and Sitti (2007); Figure 1M). A rigid tail lightly pressed against a wall can create a counter-moment (Autumn et al. (2005); Kim et al. (2008); Hawkes et al. (2011)), which helps create a more even pressure distribution on the robot’s feet and reduces the adhesion required by the front feet (Autumn et al. (2005); Unver et al. (2006); Hawkes et al. (2011); Lee et al. (2012); Estrada et al. (2014)).

RiSE can imitate a gecko’s posture by using its tail (like a bicycle’s kickstand) to regain balance and avoid falling if pitch back was unavoidable (Jusufi et al. (2008); Figure 1D). Tankbot-I (Figure 1K) uses its rigid tail as a support when transiting between vertical and horizontal surfaces (Autumn et al. (2005); Lee et al. (2012); Kalouche et al. (2014)). The length of the tail and angle between the tail and body are also important parameters for a robot with a rigid tail to avoid pitch-back. If the downward forces at the feet are equal and the front and rear feet of the robot have the same friction coefficient, then the feet with a lower normal force slide down or detach from the surface first, and the other feet slide down or detach afterward (Unver et al. (2006)). To prevent this and achieve stable climbing on a constant slope, a certain tail angle should be determined to obtain an additional torque (Lee et al. (2012)) to maintain equal normal forces for the front and rear feet (Unver et al. (2006); Unver and Sitti (2010)). Furthermore, the robot’s tail angle should be adaptively changed to provide appropriate torques for different slopes. Changing the angle of a rigid tail could also adjust the robot’s center of gravity to achieve a stable posture. A longer tail requires less adhesion to prevent the robot from falling (Menon et al. (2004); Unver et al. (2006); Jusufi et al. (2008); Hawkes et al. (2011); Estrada et al. (2014)). However, the weight of the robot increases with the tail length; a longer tail requires more space to turn, limiting the robot’s movement in small spaces (Murphy and Sitti (2007); Unver and Sitti (2010)).

### 2.2 Soft Tail

A few climbing robots are gradually adopting soft tails which are usually made of soft materials that can comply with the terrain under a certain load. Typically, the motor-driven soft tail of a slope-climbing robot (Figure 1H) progressively touches the surface from the tip of the tail and generates a preload. Compared to rigid structures, soft structures have higher flexibility, safety, and adaptability, as well as incomparable advantages in narrow space and unstructured environment operations (Majidi (2014); Gu et al. (2018); Schiller et al. (2019)). Furthermore, soft tail has a greater adhesion and a larger effective contact area, facilitating the movement of climbing robots on smooth vertical surfaces. It is also effective in terms of dissipating energy from an impact, reducing damping vibration, and counteracting discontinuous forces and motion (Nguyen et al. (2019); Siddall et al. (2021a)). In contrast, a (simple) active rigid tail can produce large inertial forces and moments with a small rotation when the tail presses against a wall (Saab et al. (2018); Unver et al. (2006)), leading to vibration and even falling. The effect can be reduced by introducing additional damping mechanisms.

Because of the undeformable nature of a rigid tail, the preload process and the surface area contacting the wall is limited by the wall. A soft tail continues to approach the wall until the entire tail is pressed against and makes complete contact with it. Moreover, its adaptability makes it inherently robust to environmental uncertainties (Kim et al. (2013)). To verify the dual fallen tree transition, Siddall et al. (2021b) used two obstacles with heights equal to the height of the robot’s wheels and separation equal to twice the length of the robot. Experiments showed that a fixed rigid tail prevented the robot from pitching when climbing, and a soft tail bent and allowed it to comply with obstacles (Siddall et al. (2021b); Figure 1J). The compliance of a soft tail can also increase the surface area contacting the wall, which is necessary to take advantage of the morphological characteristics (such as keeled subcaudal scales) of a gecko’s tail.

## 3 Discussion

For climbing robots that rely on adhesive feet to move on vertical surfaces (Menon et al. (2004); Unver et al. (2006); Kim et al. (2008); Hawkes et al. (2011); Estrada et al. (2014); Kalouche et al. (2014); Gu et al. (2018); Chen et al. (2019)), the counter-moment generated when the tail contacts the wall can transfer the load from the rear feet to the front feet and reduce the adhesion force required for the front feet to prevent pitch-back (Unver et al. (2006); Jusufi et al. (2008); Kim et al. (2008); Hawkes et al. (2011); Lee et al. (2012); Estrada et al. (2014)). For example, Schultz J. T. et al. (2021) presented a robot that could climb vertical slopes using a rigid tail and claws for adhesion with a gecko-like gait. A rigid tail can provide a large preload, but it can also generate vibrations when the tail is pressed. A soft tail is more stable but it can provide a limited preload. The tail length and Young’s modulus are proportional to the robot’s ability to prevent pitch-back (Menon et al. (2004); Unver et al. (2006); Estrada et al. (2014)). For example, after equipping the robot with a 6.6 cm tail, the maximum slope that Mini-Whegs™ 7 could climb increased from 50° to 60°, and it further improved to 90° when the tail length was 25 cm (Daltorio et al. (2006); Table 1). However, a longer tail also indicates greater weight and greater space required for turning. Thus, it is important to have a suitable tail length to body length ratio (Saab et al. (2018)). According to Table 1, the appropriate tail length to body length ratio ranges for active and passive tails are [0.34, 0.67] and [0.43, 0.74], respectively, whereas the best ratio for a fixed tail is 0.5. This implies that a passive tail requires a longer length than an active one. Analysis using finite element model (FEM) has revealed that the tail length and Young’s modulus are inversely proportional to the adhesion force required by the front feet (Menon et al. (2004)).A soft tail has a greater adhesion and effective contact area and is more adaptable to the environment (Heilmann and Rigney (1981)), making it better at moving over slippery surfaces and complex terrains (Daltorio et al. (2006)). However, a soft tail wears out easily due to frictional adhesion. To avoid pitch-back and achieve plane transition, a rigid tail can be used as a fifth limb and give a point load with a large radius/lever arm, which helps the robot’s front feet stick to the wall (Unver and Sitti (2009)). A soft tail, on the other hand, can convert the ground reaction force (GRF) into elastic deformation and provide an edge load (Siddall et al. (2021b); Santiago et al. (2016)). The addition of a tail can improve the lifting height of the abdomen of a gecko robot during vertical movement, which allows the robot to move over larger obstacles.

The motion type of the tail is another key factor for the environmental adaptability of climbing robots. A fixed rigid tail is suitable for tasks with a constant slope (Figure 1M), whereas a passive tail and a fixed soft tail are more suitable for complex terrain with gradient variations (Figure 1J). Conversely, an active tail is more suitable for special tasks such as obstacle crossing and air-righting by rotating the tail (Figure 1F). Accordingly, considering different combinations of motion type (fixed, active, and passive) and stiffness (soft and rigid) is essential in robot tail design for robustness in various situations. The body motion and gait pattern also influence the tail design. A robot with a bendable body (Haomachai et al. (2021)) may need a soft or lateral undulating tail to compensate for the body swing. A robot with a lizard-like gait pattern (raising two legs at the same time) (Shao et al. (2022)) can benefit from a tail that provides a substantial preload or functions as additional support for stable slope climbing.

The tail lateral undulation has a significant influence on a gecko’s locomotion. The undulating tail could help geckos to run across water by improving their stability and forward velocity (Nirody et al. (2018)). Losing tail motion in geckos results in a more sprawled posture for the loss compensation (Jagnandan and Higham (2017)). However, only a few gecko-inspired robots with undulating tails exist. Undulating tails are commonly used in amphibious robots inspired by lizards and salamanders (Nirody et al. (2018); Karakasiliotis and Ijspeert (2009); Kim et al. (2015); Figure 1G). Typically, each robot implements a specific tail function. Integrating multiple functions for a tail in a robot remains a challenge. In future studies, gecko-tail imitation should focus on the tail actuation system that more closely model muscle mechanics (Xiong et al. (2014); Xiong and Manoonpong (2021)) or hybrid rigid-soft structure/material. For example, soft materials could be used to realize adaptability and 3D dexterity, with rigid links or keels integrated into these soft materials to achieve structural stability (Schwaner et al. (2021)) and an adaptive bio-inspired muscle model can be applied for active tail-joint compliance control with online adaptation (Huerta et al. (2020)).

## 4 Conclusion

The tail of a climbing robot is important in terms of improving the maximum climbing angles and avoiding slipping or pitch-back (Jusufi et al. (2008); Kim et al. (2008); Chen et al. (2019)). A longer tail requires less adhesion force for the front feet of the robot to adhere to the substrate (Menon et al. (2004); Unver et al. (2006); Hawkes et al. (2011)); however, the tail length is limited by practical and aesthetic factors. Both rigid and soft tails have advantages and disadvantages. A soft tail is more robust and stable and can more effectively reduce the required adhesion force, while providing a good preload in an unstructured environment. In contrast, a rigid tail can better adjust the robot’s center of gravity, provide a large preload, and aid in plane transition, and it is less prone to wear. The weight of the robot, substrate surface, and environment are the main factors that should be considered when choosing the material and motion type for a robot’s tail. Finally, adding a gecko-inspired tail can help with self-righting and increase locomotion performance in both horizontal and vertical planes.
